# HPV and vaginal microecological disorders in infertile women: a cross-sectional study in the Chinese population

**DOI:** 10.1186/s12985-022-01869-0

**Published:** 2022-08-25

**Authors:** Li Wang, Lin He, Junyu Chen, Shuyao Wei, Hongzhou Xu, Mengjun Luo

**Affiliations:** Department of Clinical Laboratory, Chengdu Women’s and Children’s Central Hospital, School of Medicine, University of Electronic Science and Technology of China, No. 1617 Ri Yue Street, Chengdu, 611731 Sichuan China

**Keywords:** Infertility, Human papillomavirus, Vaginal microecological

## Abstract

**Background:**

The purpose of this study was to evaluate the distributions of vaginal microbiome dysbiosis and human papillomavirus (HPV) subtypes in infertile women and explore the correlations of HPV infection and vaginal microbiome dysbiosis with infertility.

**Methods:**

In total, 1464 women aged 18–50 years were included in this study; 649 participants were included in the infertility group, and 815 participants were included in the normal group. The participants were tested for HPV, and their vaginal microecology was examined. The χ^2^ test and Spearman regression were used for statistical analysis, and binary logistic regression was performed to identify the risk factors for infertility.

**Results:**

The patients in the infertility group were younger than those in the normal group, and the proportions of bacterial vaginosis and vaginal imbalance in the infertility group were significantly higher than those in the normal group. The incidence proportions of high-risk HPV types in the infertility group were significantly higher than those in the normal group, and the proportions of high-risk subtytes HPV16, HPV39, HV52, HPV56, and HPV68 were significantly higher in the infertility group than in the normal group. However, there were no significant differences in the incidences of low-risk HPV types. The incidence proportions of vaginal flora imbalance and HPV infection in the infertility group were significantly higher than those in the normal group. HPV16, HPV33, HPV51, HPV52and HPV58 infections were independent risk factors for infertility.

**Conclusions:**

Vaginal microecological imbalance and HPV infection are directly related to infertility, and precautions should be taken.

## Background

Human papilloma virus (HPV) is a small DNA tumour virus that belongs to the Papillomavirus family, with more than 200 types identified [1]. It is estimated to be the most common sexually transmitted virus. According to statistics, in women, the probability of viral infection within the lifetime is more than 80%, and in men, it is more than 90%. Although most people with HPV infection are asymptomatic, persistent HPV infection can lead to cervical cancer, genital warts and other diseases. Therefore, HPV infection has attracted increasing attention [2, 3]. HPV and *Chlamydia trachomatis* have relatively high incidence proportions, and the peak age at infection in females is approximately 20 years old [4, 5]. There are many influencing factors, such as a larger number of sexual partners. Most infected patients lack typical clinical symptoms, and more than 90% of HPV infections are transient, meaning they will be cleared by an incompletely understood immune response within 6–18 months [6]. Reinfection with the same or a different HPV subtype may occur [7]. Therefore, an infection that has not been identified and treated can result in persistent infection and continued transmission in the population. Persistent HPV infection can lead to serious complications, and approximately 20% of infections with high-risk HPV genotypes will develop into cervical intraepithelial neoplasia or cervical cancer within 5 years [8, 9].

Recent studies have shown that persistent HPV infection may affect human health, including reproductive dysfunction and pregnancy outcomes of abortion and preterm birth [10]. Because of the adverse effects of persistent viral infection in women; the harmful effects of HPV on male sperm quality, including reduced sperm motility and the production of antisperm antibodies; and the adverse effects of vertical transmission in early embryos, HPV infection may be the cause of infertility in couples [11–13]. In animal experiments, HPV infection changed the expression of developing blastocysts, leading to DNA breakage and trophoblast cell apoptosis [14]. In recent years, the incidence of infertility has remained very high. It is a complex human health problem that can seriously affect quality of life. In 2010, the incidence proportion of infertility reached 12.4%, and the incidence proportion of infertility is even higher in southern Asia, Africa and central Asia [15]. In a study evaluating the outcomes of HPV infection and in vitro fertilization (IVF), they found that the proportion of pregnancy among those with HPV infection was lower than that among those without HPV infection [16].

The vaginal microbial environment is directly related to HPV infection [17]. Lactobacilli are the main flora constituents of the vaginal microbial community. These organisms produce lactic acid, bacteriocin and biosurfactants to resist infection by pathogenic bacteria by adhering to mucous membranes, providing extensive protection [18, 19]. However, whether vaginal microecological disorder caused by microecological imbalance and HPV infection is directly related to the occurrence of infertility and whether infection with the abovementioned pathogens will progress to long-term complications have not been reported thus far. In this study, associations of the vaginal microbiota structure and HPV infection with the occurrence of infertility were assessed to identify effective strategies to prevent infertility.

## Materials and methods

### Study population

This study included 649 infertile women diagnosed in the reproductive medicine centre and 815 healthy women of childbearing age from the Department of Gynaecology at Chengdu Women’s and Children’s Central Hospital, School of Medicine, University of Electronic Science and Technology of China, from January 2019 to December 2021. The infertility group was defined as those with regular sexual intercourse without the use of any contraceptive measures for at least 1 year without conception. The inclusion criteria for the control group were menstrual regularity, married, natural pregnancy, and age between 18 and 50 years. Participants were recruited by doctors and no use of hormones, antibiotics, or immunosuppressive agents in the past 2 weeks; no sexual intercourse; and no history of vulva/vaginal medication use in the past 3 days. All the vaginal and cervical swab samples were collected by professional doctors. First, the cervix was exposed with a vaginal speculum, and a cervical brush was placed on the cervix to obtain a sufficient number of epithelial cells, which were placed in an elution tube. A second vaginal secretion sample was collected with a sterile long-handle swab, which was used for smear examination and to determine the pH value and H_2_O_2_ concentrations of the vaginal secretion. All procedures and protocols applied in this study were approved by the ethics committee of the Women and Children Affiliated Hospital of the Medical College of the University of Electronic Science and Technology (Grant No. B2019(1)) and the informed consent of the research subjects has been obtained.

### Microecological detection

Vaginal smears were subjected to Gram staining [20]. After Gram staining, vaginal cleanliness, white blood cells, Lactobacillus number, *Candida* spp. (spores, blastospores, and pseudohyphae), *Trichomonas vaginalis*, clue cells, bacterial density, flora diversity and dominant bacteria were observed. The observations followed the guidelines of the National System for External Quality Assessment (NSEQA) and College of American Pathologists (CAP) [21].

The diagnostic criteria for bacterial density, vaginal health, *Trichomonas* vaginitis (TV), vulvovaginal candidiasis (VVC) and bacterial vaginosis (BV) referred to references [22–25]. Bacterial density and flora diversity are reported according to the reference literature [26]. The density of bacteria refers to the density and distribution of microhabitats in a specimen. In this study, bacterial density was observed under the optical microscope and classified into 4 grades: grade I (1 +): 1–9; grade II (2 +): 10–99; grade III (3 +): 100 and above; and grade IV (4 +): bacterial clusters in the full visual field. Flora diversity refers to the number of all types of bacteria in the microscope field of view under high magnification (1000 times). The diversity of flora was divided into four grades: grade I (1 +): 1–3 flora species; grade II (2 +): 4–6 flora species; grade III (3 +): 7–10 flora species; and grade IV (4 +): more than 10 flora species. According to the Expert Consensus on the Clinical Application of Vaginal Microecosystem Assessment of the Cooperative Group of Infectious Diseases in the Department of Obstetrics and Gynecology of the Chinese Medical Association [27], a normal vaginal microecosystem was defined as grade I vaginal health, level II–III bacterial density, level II–III flora diversity, and Lactobacillus spp. as the dominant bacterial species. Additionally, the production of H_2_O_2_ was normal, and the vaginal pH value was 3.8–4.5. When any of the above indicators (such as vaginal health, bacterial density, flora diversity, dominant bacteria, pH value or Lactobacillus abundance) were abnormal, microecological dysbiosis was diagnosed.

### HPV genotyping detection

Exfoliated human cervical cell samples were collected, and HPV genotyping was performed by using a human papillomavirus genotyping diagnosis kit with a gene chip (Genetel Pharmaceuticals, Shenzhen). The method detected 24 HPV nucleic acid subtypes, including those of high-risk HPV subtypes 16, 18, 31, 33, 35, 39, 45, 51, 52, 53, 56, 58, 59, 66, 68, 73, 82, and 83 and low-risk HPV subtypes 6, 11, 42, 43, 44, and 81. The DNA was extracted from the exfoliated human cervical cell samples by the isolation reagent recommended by the kit and was extracted according to the manufacturer’s instructions. A Roche Lightcycler 480 nucleic acid detector was used for nucleic acid amplification and PCR amplification of DNA samples with primers, PCR reactions. PCR was performed for each experimental sample, and the reaction mixtures consisted of 2 μl of DNA, 27.6 μl of HPV PCR mix (including specific primers), and 0.4 μl of Taq/UNG (final volume: 30 μl). The PCR conditions were 1 cycle of hot-start activation at 95 °C for 10 min; followed by 40 cycles of denaturation at 95 °C for 30 s, annealing at 52 °C for 30 s, and extension at 65 °C for 30 s; and a final extension at 65 °C for 5 min. Then, specific hybridization of the amplified products was performed with an HPV-type gene chip. The chip detection reading system (HPV-GenoCam-9600) scans the chip image and data analysis after hybridization and finally obtains the HPV virus gene detection and typing results.

### Statistical analysis

The data were analysed with SPSS statistical software SPSS 22.0 (IBM, Chicago, USA). The χ2 test was used to analyse the differences in microecological factors and HPV detection proportions between groups. *P* < 0.050 indicated that the difference was statistically significant. Spearman correlation coefficients were computed to examine the association between HPV and infertility. Multivariate logistic regression analysis was used to predict multiple indicators for infertility.

## Results

### Study population

The general data of the 1464 pregnant women are shown in Table 1. Their ages ranged from 18 to 50 years old. There were 649 participants in the infertility group and 815 participants in the normal group. The participants were divided into groups according to age, namely, 18–30 years old and 31 to 40 years old, and there was a significant difference in age between the infertility group and the normal group. (*P* < 0.001, shown in Table 1). However, there was no difference in those aged 41–50 years between the groups. The average age of the infertility group (mean ± SD, 28.55 ± 3.16) was significantly lower than that of the normal group (mean ± SD, 30.13 ± 4.47), *P* < 0.001. The prevalence of previous pelvic inflammation was significantly higher in the infertility group than in the normal group, but there was no significant difference in the proportion of *Chlamydia trachomatis* infection between the two groups (shown in Table 1).

**Table 1:** General data of the 1464 included women

Variables	Infertility group	Normal group	*χ*^2^	*P*
	Cases (%)	Cases (%)		
18–30 (years)	339 (52.23)	579 (71.04)	54.65	< 0.001
31–40 (years)	292 (44.99)	224 (27.49)	48.52	< 0.001
41–50 (years)	18 (2.78)	12 (1.47)	3.05	0.081
Previous pelvic inflammatory disease	91 (14.02)	48 (5.89)	27.80	< 0.001
Previous or/and current Chlamydia infection	51 (7.86)	42 (5.15)	4.44	0.035

### Comparison of vaginal microecology between the infertility and normal groups

There were 154 cases of BV in the infertility group, accounting for 23.73% of the population, which was significantly higher than 89 (10.92%) in the normal group (*χ*^2^ = 42.818, *P* < 0.001). There was no significant difference in the prevalence of VVC or TV between the two groups. However, the proportion of vaginal disorders (including abnormal vaginal health, bacterial density, flora diversity, dominant bacteria, pH value and Lactobacillus abundance) in the infertility group was significantly higher than that in the normal group (*χ*^2^ = 78.14, *P* < 0.001, Table 2).

**Table 2:** Analysis of difference in pathogenic microorganisms and vaginal microecology in infertility and normal groups

Microecological factors	Infertility group	Normal group	*χ*^2^	*P*
	Cases (%)	Cases (%)		
BV	154 (23.73)	89 (10.92)	42.82	< 0.001
VVC	20 (3.08)	37 (4.54)	2.05	0.152
TV	10 (1.54)	6 (0.74)	2.16	0.141
Vaginal dysbiosis	392 (60.40)	303 (37.18)	78.14	< 0.001

### HPV infection in the infertility and normal groups

The number of women with HPV infection in the infertility group was 247 (38.06%), and the number of women with HPV infection in the normal group was 181 (22.21%). Both the infertile group and the normal group had higher infection proportions of high-risk HPV subtypes than low-risk HPV subtypes. The proportions of infection with high-risk HPV subtypes and multiple infections in the infertile group were significantly higher than those in the normal group (*P* < 0*.*001). However, there was no significant difference in the proportion of infection with low-risk HPV subtypes between the infertile group and the normal group (*P* > 0.050, Table 3). The number of patients with multiple infections with more than two types of HPV in the infertility group was significantly higher than that in the normal group (*P* = 0*.*004, Table 3).

**Table 3:** Analysis of difference in HPV infection in infertility and normal groups

Types	Infertility group	Normal group	*χ*^2^	*P*
	Infections cases (%)	Infections cases (%)		
Low risk	39 (6.01)	41 (5.03)	0.67	0.413
High risk	208 (32.05)	140 (17.18)	44.10	< 0.001
Multiple infections	47 (7.24)	31 (3.80)	8.47	0.004

### Distributions of HPV subtypes in the infertility and normal groups

Among the six low-risk HPV subtypes, the proportion of HPV81 infection was the highest, and the proportion of HPV81 infection in the infertile group was higher than that in the normal group (*P* = 0.049). Among the 18 high-risk HPV subtypes, the proportion of HPV52 infection was the highest, followed by the proportions of HPV16, HPV58 and HPV56 infections. Among the subtypes with higher infection proportions, the proportions of HPV16, HPV39, HPV52, HPV56 and HPV68 infections were significantly higher in the infertile group than in the normal group. The difference was statistically significant (*P* < 0*.*005, Table 4). The proportions of high-risk HPV82 and HPV83 infection were lower.

**Table 4:** Analysis of difference in distribution of HPV subtypes in infertility and normal groups

Infection type	HPV genotype	Infertility group	Normal group	*χ*^2^	*P*
		Infections cases (%)	Infection cases (%)		
Low risk	HPV6	3 (0.46)	8 (0.98)	1.31	0.253
	HPV11	2 (0.32)	1 (0.12)	0.61	0.436
	HPV42	7 (1.08)	9 (1.10)	0.00	0.963
	HPV43	4 (0.62)	5 (0.61)	0.08	0.782
	HPV44	6 (0.92)	8 (0.98)	0.01	0.920
	HPV81	17 (2.62)	10 (1.23)	3.87	0.049
High risk	HPV16	37 (5.24)	11 (1.35)	21.57	< 0.001
	HPV18	11 (1.69)	6 (0.74)	2.89	0.089
	HPV31	3 (0.46)	4 (0.49)	0.01	0.937
	HPV33	7 (1.08)	3 (0.37)	2.69	0.101
	HPV35	4 (0.62)	2 (0.25)	1.22	0.270
	HPV39	15 (2.31)	8 (0.98)	4.13	0.042
	HPV45	1 (0.15)	1 (0.12)	0.03	0.872
	HPV51	12 (1.85)	12 (1.47)	0.32	0.573
	HPV52	41 (6.32)	33 (4.05)	3.87	0.049
	HPV53	10 (1.54)	17 (2.09)	0.59	0.441
	HPV56	15 (2.31)	6 (0.74)	6.34	0.012
	HPV58	21 (3.24)	14 (1.72)	3.57	0.059
	HPV59	10 (1.54)	10 (1.23)	0.26	0.607
	HPV66	11 (1.69)	8 (0.98)	1.44	0.231
	HPV68	11 (1.69)	5 (0.61)	3.91	0.048
	HPV73	2 (0.31)	0 (0)	2.52	0.113
	HPV82	0 (0)	0 (0)	-	-
	HPV83	0 (0)	0 (0)	-	-

### HPV infections in women in different age groups in the infertility and normal groups

The HPV infection proportions in those aged 18–30 and 31–40 years were higher in the infertility group than in the normal group (*P* < 0.001, Table 5), and there was no significant difference in the HPV infection proportion between the groups in 41- to 50-year-old women (*P* > 0.050). Compared with that in the control group, the total HPV infection proportion in the infertility group was significantly higher (*P* < 0.050, Table 5).

**Table 5:** Analysis of differences in HPV infection between the infertile and normal groups of with different ages

Age (years)	Infertility group		Normal group		*χ*^2^	*P*
	n	Infections cases (%)	n	Infections cases (%)		
18–30	339	93 (27.43)	579	85 (14.68)	22.25	< 0.001
31–40	292	81 (27.74)	224	39 (17.41)	7.56	0.006
41–50	18	7 (38.89)	12	3 (25.00)	0.63	0.429
Total	649	181 (27.89)	815	127 (15.58)	32.94	< 0.001

### The relationship between vaginal microecology and HPV infection

In the infertility group, the proportion of HPV positivity in patients with vaginal dysbiosis was significantly higher than that in the normal group (*χ*^2^ = 12.02, *P* = 0.001, Table 6). However, there was no difference in the proportion of HPV positivity in patients with normal vaginal microecology between the infertility group and the normal group (Table 6).

**Table 6:** Vaginal microbiome and HPV infection proportions in infertile and normal groups

Vaginal microecology	Infertility group		Normal group		*χ*^2^	*P*
	n	HPV infection cases (%)	n	HPV infection cases (%)		
Vaginal dysbiosis	392	168 (42.86)	303	91 (30.03)	12.02	0.001
Normal vaginal microecology	257	13 (5.06)	512	36 (7.03)	0.09	0.296

### Correlation analysis

There was a significant positive correlation between age and HPV infection (r = 0.073, *P* = 0.005), but there was no correlation between age and vaginal microecology imbalance (r = −0.028, *P* = 0.227). Vaginal microecology balance showed a significant negative correlation with HPV infection (r = −0.123, *P* = 0.001).

### HPV genotypes as independent risk factors for infertility

After adjustment for age, HPV16 (OR 3.296, 95% CI 1.507–7.213, *P* = 0.003), HPV33 (OR 9.972, 95% CI 1.196–83.131, *P* = 0.034), HPV51 (OR 0.313, 95% CI 0.146–0.673, *P* = 0.003), HPV52 (OR 3.665, 95% CI 1.960–6.853, *P* < 0.001) and HPV58 (OR 2.364, 95% CI 1.043–5.357, P = 0.039) infections were independent risk factors for infertility.

## Discussion

In recent years, with the increasing incidence of sexually transmitted diseases and the increasing number of operations in the uterine cavity, the incidence of female infertility has increased, and it has become a worldwide problem in women of childbearing age [28], affecting approximately 10–30% of couples [29, 30]. The American College of Obstetricians and Gynecologists (ACOG) defines infertility as the failure to achieve pregnancy within 12 months of unprotected intercourse or therapeutic donor insemination of women less than 35 years or within 6 months in women aged over 35 [31]. It has been reported in the literature that persistent infection with high-risk HPV may be associated with female infertility [32]. HPV is one of the most common sexually transmitted pathogens, and infection is obviously age-related; infertile women are in the peak age range of HPV infection [33, 34]. There are many reasons for infertility, and reproductive tract infection is one important reason. Our study confirmed that infertile patients were younger than normal reproductive-aged women. The incidence proportions of previous pelvic inflammation and mycoplasma infection in infertile women were significantly higher than in normal reproductive-aged women. Pelvic inflammatory disease and associated complications are causes of infertility. Fallopian tube epithelial cell inflammation caused by pelvic inflammatory disease causes obstruction to varying degrees, leads to irreversible changes, and further leads to infertility. The blockage and inflammation of the oviduct are often caused by retrograde infection of the reproductive tract. In our study, the proportions of BV and vaginal disorders in the infertility group were significantly higher than those in the normal group, and there was no significant difference in the proportions of fungal and *Trichomonas vaginalis* infections between groups. Vaginal microecology imbalance seems to be a potential driver of HPV infection [35, 36]. In this study, the proportion of HPV infection in patients with vaginal microecological disorder in the infertility group was higher than that in the normal group. To some extent, the above relationship reveals a relationship between vaginal microecological disorders and inflammatory diseases and infertility.

Worldwide, infertility caused by infection with *Trichomonas vaginalis*, *Chlamydia trachomatis*, and other microorganisms is widely researched. Although the diagnosis and treatment of *Chlamydia trachomatis* and *Neisseria gonorrhoeae* have been confirmed to prevent the occurrence of infertility, we still need to identify additional factors that affect infertility [37, 38]. HPV genotype, especially high-risk HPV genotypes, is an important factor affecting infertility and placental dysfunction. In trophoblast cells transfected with plasmids containing the HPV16 genome, the proportion of apoptosis was 3 to 6 times that of trophoblast cells transfected with empty plasmids. Moreover, apoptosis may lead to placental dysfunction and a reduced ability of the embryo to implant in the uterine wall [39]. At the same time, there are reports in the literature that HPV can reduce endometrial implantation of trophoblast cells, thereby increasing the risk of miscarriage [40]. HPV DNA was detected more frequently in spontaneous abortions. In a study of 108 miscarriage patients, HPV16 and HPV18 DNA were detected in 7.4% of embryos [41]. In our study, the proportion of high-risk HPV infection in the infertility group was significantly higher than that in the normal group, and the mixed HPV infection proportion in the infertility group was also higher than that in the normal group. We further classified the types of HPV infection in the two groups and found that only HPV81 was different among the low-risk types, while the proportions of high-risk HPV16, HPV39, HPV52, HPV56 and HPV68 infections were higher than those in the normal group. HPV infection rates vary greatly among different regions, but HPV16 is highly prevalent among women in many countries worldwide. HPV16 infection is related to infertility and is also the main type of infection leading to cervical cancer. In Asia and China, HPV16 and HPV52 are the main types of infections [42]. At present, there are few reports on the exact relationship between HPV16 infection and infertility, but most studies suggest that HPV infection may be one of the risk factors for infertility. Our conclusion also confirmed that the vast majority of HPV infections in infertility patients were high risk subtypes. Therefore, high-risk HPV screening in women of childbearing age and early vaccination may play roles in the prevention of infertility.

Although the HPV incidence has been increasing worldwide and HPV is the most common sexually transmitted disease, most patients are asymptomatic. In the past, most research focused on whether HPV was responsible for infertility and its impact on assisted reproductive technology. It is important to assess the types of HPV leading to infertility and to identify risk factors for infertility [11]. In our study, we confirmed that age and HPV infection were positively associated with infertility and that vaginal flora imbalance was negatively correlated with infertility. Age was an important risk factor for infertility, consistent with the literature. With increasing pressure at work and in life, many people are forced to marry and have children late. With increasing age, an increasing number of follicles undergo apoptosis. At the age of 30, egg reserved in the ovaries can reach 12, but at the age of 40, only 3% of egg reserves remain, and decreased hormone levels may lead to infertility HPV infection was also positively correlated with infertility, so we further identified the independent risk factors of infertility. We determined that HPV16, HPV33, HPV51, HPV52 and HPV58 infections were independent risk factors for infertility. Our conclusions are basically consistent with the eight HPV genotypes with the highest incidence in the United States, Europe and Africa: HPV16, 18, 31, 33, 35, 45, 52, and 58 [43]. This data will provide a reference for HPV disease screening, treatment and vaccine development.

Multiple infections of HPV will lead to changes in the vaginal microenvironment. Moreover, it is more difficult and time-consuming to clear multiple infections than single infections, and changes in the normal vaginal environment can easily lead to infections by other viruses, bacteria, mycoplasma, and chlamydia as well as coinfections [17]. Multiple factors are likely to cause chronic inflammation in the uterus and fallopian tubes and eventually lead to infertility. Therefore, screening for HPV infection and examination of vaginal microecology should be strengthened among women of childbearing age to prevent infertility caused by HPV infection and changes in the vaginal environment.

## Conclusion

Our research showed that HPV and vaginal microecological disorders were related to infertility, and some types of high-risk HPV subtypes were especially high-risk factors for infertility. However, the small sample size and lack of data on infection mechanisms are limitations of this study. A study with a larger sample size is needed to further elucidate the mechanisms of the effects of HPV and vaginal microecology on infertility.

## Data Availability

The authors confirm that all the data based findings are fully available without restriction. All relevant data are included in the paper and references. The datasets used and/or analysed during the current study available from the corresponding author on reasonable request.
